# Practicing doctors' perceptions on new learning objectives for Vietnamese medical schools

**DOI:** 10.1186/1472-6920-7-19

**Published:** 2007-06-28

**Authors:** Luu Ngoc Hoat, Do Van Dung, E Pamela Wright

**Affiliations:** 1 Biostatistics and Medical Informatics Department, Faculty of Public Health, Hanoi Medical University, Dong Da, Hanoi, Vietnam; 2 Biostatistics and Medical Informatics Department, Faculty of Public Health, Ho Chi Minh City Medical and Pharmaceutical University, Ho Chi Minh City, Vietnam; 3 Medical Committee Netherlands – Vietnam, Dong Da, Hanoi, Vietnam

## Abstract

**Background:**

As part of the process to develop more community-oriented medical teaching in Vietnam, eight medical schools prepared a set of standard learning objectives with attention to the needs of a doctor working with the community. Because they were prepared based on government documents and the opinions of the teachers, it was necessary to check them with doctors who had already graduated and were working at different sites in the community.

**Methods:**

Each of the eight medical faculties asked 100 practising recent graduates to complete a questionnaire to check the relevance of the skills that the teachers considered most important. We used mean and standard deviation to summarize the scores rated by the respondents for each skill and percentile at four points: p50, p25, p10 and p5 to describe the variation of scores among the respondents. Correlation coefficient was used to measure the relationship between skill levels set by the teachers and the perception of practicing doctors regarding frequency of using skills and priority for each skill. Additional information was taken from the records of focus group discussions to clarify, explain or expand on the results from the quantitative data.

**Results:**

In many cases the skills considered important by teachers were also rated as highly necessary and/or frequently used by the respondents. There were, however, discrepancies: some skills important to teachers were seldom used and not considered important by the doctors. In focus group discussions the doctors also identified skills that are not taught at all in the medical schools but would be needed by practising doctors.

**Conclusion:**

Although most of the skills and skill levels included in the learning objectives by the teachers were consistent with the opinions of their graduates, the match was not perfect. The experience of the graduates and their additional comments should be included as inputs to the definition of learning objectives for medical students.

## Background

When a medical curriculum is going to be updated, practicing physicians can provide highly relevant recommendations on how to improve the teaching to prepare the graduates better for their future work [1,2]. If they are asked to provide feedback within a few years of their graduation, they will remember what they learned and compare it to what they needed to know as they began to practice medicine [3].

During the past several years, the medical schools in Vietnam have been revising their curriculum to make it more appropriate to the conditions where graduates will be likely to work. Since the early 1990s, the Vietnamese Government has had a policy to train doctors to be more community- than hospital-oriented. The government also set a target for the year 2000, to have at least 40% of commune health centres staffed by a medical doctor. These policies put pressure on medical schools to revise their curriculum and teaching methods so that graduates would function better at community level. Of the ten medical schools in Vietnam, eight fall under the Ministry of Health and the Ministry of Education and Training. Eight are regional: Thai Nguyen, serving the northern midlands and mountainous areas; Hai Phong, serving the Red River Delta and Northern provinces; Thai Binh, serving the provinces of the Red River Delta and Northern provinces of central region; Hue, serving the central region; Tay Nguyen, serving the Central Highlands and the central coastal provinces; and Can Tho, serving the Mekong Delta. The larger medical universities of Hanoi and Ho Chi Minh City (HCMC) provide undergraduate and postgraduate training for the whole country. These eight schools worked together in a project to improve their teaching and to make it more community-oriented. Standard learning objectives were defined using a participatory process among senior teaching staff in the eight schools. The development of these objectives was based on a set of 274 health problems and solutions prioritized by a combination of national and regional health statistics and the opinions of senior teachers in the eight schools [4].

These learning objectives were prepared as a book listing the knowledge, attitudes and skills (KAS Book) that a graduating doctor from any medical school in Vietnam is expected to have. Such books have been developed in other countries, for example, the Blueprint Book in the Netherlands [5] and the Swiss Catalogue of Learning Objectives [6], usually using a Delphi approach in which key experts define the content. In the Vietnamese process, four sources of information were used to develop the KAS book [4]: (i) input from more than 1000 teachers from the eight medical schools as well as from other medical experts, (ii) review of government policies, regulations and existing study reports on training needs and health care need assessments, (iii) input from the feedback of graduating students on the value of skills they learned during six years of training, and (iv) community consultation, including newly graduated doctors already working in the community and their employers, as well as patients and their family members. This is the first time in Vietnam that practicing doctors have been consulted during a process of curriculum development.

This paper presents the results of the main part of the community consultation, the perception of recently graduated doctors about the appropriateness of the proposed learning objectives. The results from the consultation with the other community stakeholders and graduating students will be presented in separate papers. The objective was to check with practicing doctors whether the skills and the level of each skill as identified by the teachers for the learning objectives were in fact the skills that a young practicing doctor would need and often use during their daily work. Although there was general agreement between the teachers' assignment of required levels of skill learning and the importance of those skills in the practice of young doctors, there were discrepancies in several areas that will require attention in the revision of the learning objectives in future.

## Methods

### Study design

The data were collected using a cross-sectional survey among nearly 800 recently graduated doctors. Development of the data collection tools, data entry and analysis was led by Ho Chi Minh City (HCMC) Medical School, with contributions from each of the others. All eight medical schools collected data in provinces belonging to their catchment area, using the same criteria and tools. The study protocol was reviewed and approved by the Scientific Committees in each of the eight faculties; they are responsible for both technical and ethical aspects of all studies in their institutions.

### Study participants

Since more than 80% of medical school graduates in Vietnam are government employees, we selected medical doctors working in the public sector (curative or preventive branches) who had graduated within the past seven years. Doctors with postgraduate degrees were excluded, because the extra years of study would make it difficult to identify the knowledge and skills learned during undergraduate study. The allocation of medical positions by the government is homogeneous among the provinces, so each school was asked to collect data from at least 100 of its recent graduates now working in two of the six to ten provinces in their catchment area. The provinces were selected randomly by lottery. Hanoi and HCMC cities were not included in the list of eligible provinces because in these two large conurbations, the priorities in the appointment of newly qualified doctors differ from the others in that a large proportion of the doctors work in national or regional level hospitals and recent graduates always work under close supervision of more experienced and more highly qualified doctors. The survey was conducted at the health service offices either at provincial or district level. All of the respondents were asked to give informed consent verbally when they were invited to join the survey. More than 98% of invited doctors agreed to complete the questionnaire, making a total of 797 respondents. Among the respondents, random numbers were used to select members for participation in focus group discussions.

### Data collection tools

**Figure 1:**
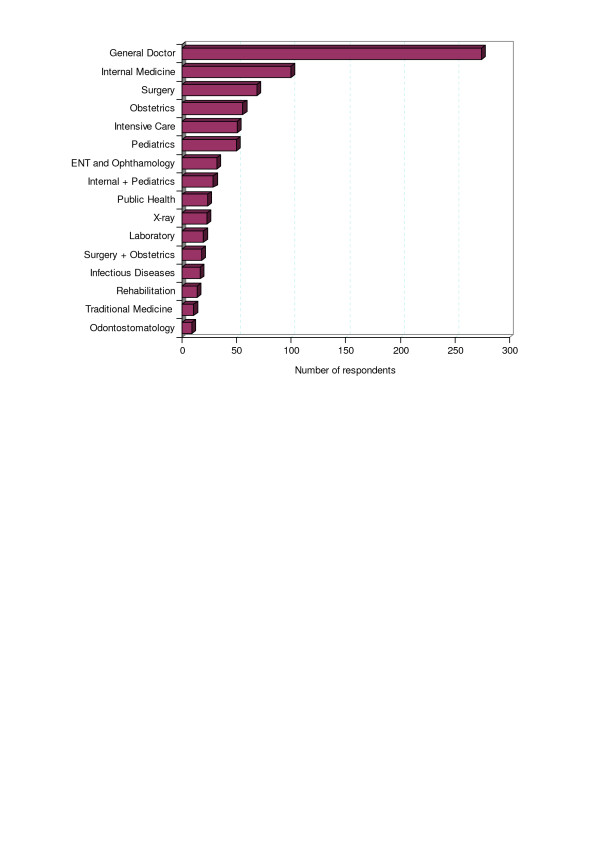
Distribution of specialities among the respondents.

We asked each doctor to complete a questionnaire to give details of their training, when they graduated, their years of working experience, current position and area of specialization. They completed a standardized data collection form (example shown in Figure 1) listing 114 skills out of the 557 related to the 274 health issues in the proposed KAS book. Each skill proposed in the KAS book was set at one of three levels for expected competency upon graduation (level 1, 2 or 3). For level 3, graduates are expected to be able to perform the skill confidently and without supervision, while for level 1, they would need close supervision; level 2 skills are intermediate.

The selection of the 114 skills included in the survey was based on the following reasoning. The study focused more on skills set at levels 2 and 3 because the teachers had identified them as the most important to teach well. It was assumed that students would have studied harder and practiced more to become competent in skills set at level 3 and level 2, than for level 1, both because their teachers gave those skills importance and because they would encounter them more often during their clinical and other training. We therefore included all of the 13 level 3 skills, and randomly selected 25% of the 290 level 2 skills and 12.5% of the 254 level 1 skills. Using this selection process, we assumed that the 114 selected skills would be representative of the 557 skills in the KAS book. For this data analysis, we grouped the skills either as 'general', for skills such as taking a patient history that most doctors in most kinds of work would use, or as belonging more closely to one of the 18 discipline-based departments involved in the project.

Using the data collection tool shown in Table 1, we first asked the doctors to report how often they used each skill in their daily work, on a scale from 0 (never used) to 6 (used at least three times daily). Secondly, we asked them to rate, on the basis of their knowledge and experience, the priority these skills would have for a general doctor working in Vietnam, on a scale from 0 (no priority) to 4 (high priority).

**Table 1:** Example of data collection tool showing 2 of the 19 disciplines

Discipline	Code^(*)^	Name of skill according to discipline	Frequency^(**)^								Priority^(***)^				
			0	1	2	3	4	5	6		0	1	2	3	4

Discipline 1 (e.g. Basic)	4	Skill 1	0	1	2	3	4	5	6		0	1	2	3	4
	8	Skill 2	0	1	2	3	4	5	6		0	1	2	3	4
	....	..........													
Discipline 2 (e.g. Internal Medicine)	134	Skill	0	1	2	3	4	5	6		0	1	2	3	4
	58	Skill	0	1	2	3	4	5	6		0	1	2	3	4
	....	..........													
Discipline 3, etc.	....	..........													

* Note:

^(*) ^Code of skill mentioned in the KAS book

^(**) ^Frequency: was categorized into seven scores:

score = 0: never used,

score = 1: used 1–2 times per year

score = 2: used 1–2 times per three months

score = 3: used 1–2 times per month

score = 4: used 1–3 times per week

score = 5: used 1–2 times per day

score = 6: used ≥ 3 times per day

^(***) ^Priority: was categorized into five scores:

score = 0: no priority

score = 1: minimum priority

score = 2: low priority

score = 3: moderate priority

score = 4: high priority

### Qualitative data

To obtain complementary qualitative data, each school also conducted four focus group discussions (FGD), two that each included ten doctors working in curative medicine and two that each included ten doctors working in preventive medicine. All of the focus group discussions used the same guidelines; the moderators, already experienced in qualitative research, were trained together to use these guidelines. The discussions took place after the respondents had completed the data collection tool, to give them the opportunity to add comments and suggestions, including any needs for additional skills that were not mentioned in the data collection form and/or the KAS book.

### Data analysis

Each of 114 selected skills was rated by all 797 respondents on the frequency of use and priority of that skill. We used means and standard deviations to summarize the scores rated by the respondents for each skill as well as for all skills assigned to each discipline. We also used percentiles at four points, p50, p25, p10 and p5, to describe the variation of scores among the respondents. Correlation coefficients were used to measure the relationship between the skill levels set by the teachers and the frequency of using skills and priority for each skill as perceived by the practicing doctors.

Additional information was taken from the records of the focus group discussions to clarify, explain or expand on the results from the quantitative data and to identify from respondents' experience any skills not yet listed by the teachers in the KAS book.

## Results

### Key characteristics of the study population

The survey obtained responses from 797 recently graduated general medical doctors, close to 100 from each of the eight faculties. The majority of the responses (75%) were from recently graduated doctors (within the past five years). Although all graduated as general medical doctors, only 34% were still working as general doctors. The others were working in the different areas of specialization shown in Figure 1. A number of specializations that are common in developed countries (such as Oncology, Neurology, Rheumatology, Cardiology) are under-represented in Vietnam and were grouped with Internal Medicine.

To facilitate the analysis of which doctors were using which skills, we assigned the respondents according to Vietnamese custom to one of five groups: 'General Medicine' including all non-specialist physicians (34% of respondents), 'Internal Medicine' including Internal Medicine, Paediatrics and Infectious Diseases (25%), 'Surgery' including Surgery and Obstetrics and Gynaecology (18%), 'Public Health' including Epidemiology, Nutrition and Food Safety, Health Management, Health Education and Environmental Health, (3%) and others.

**Figure 2:**
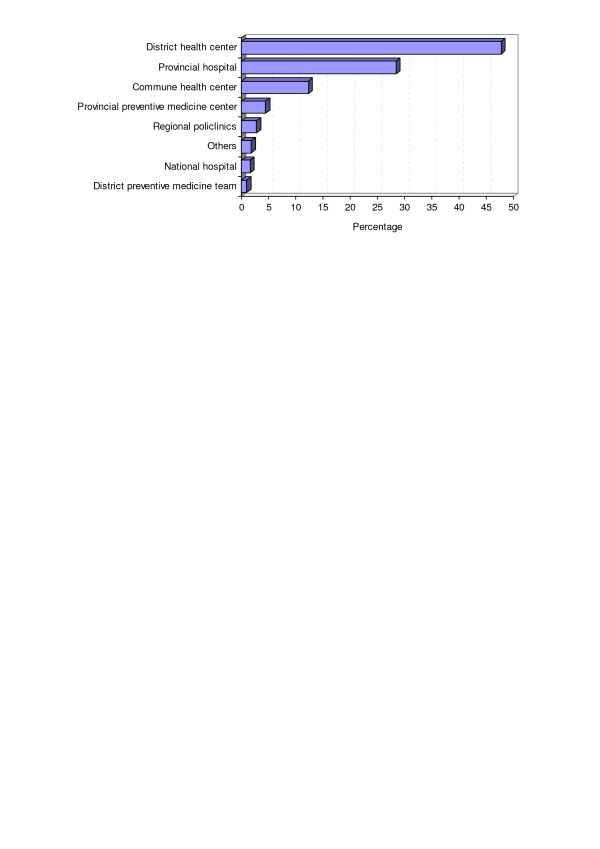
Working environment of the study participants.

Nearly half of the respondents were working in district health centres, with another quarter working in provincial hospitals (Figure 2). More than 90% were doing hospital-based clinical work. About 12% of the respondents were working at grassroots level, in commune health centres, but very few were working in preventive medicine, at either provincial (5%) or district (1%) level.

### Relevance of skill levels set by teachers and perception of the practising doctors

Each skill was assessed using three criteria: the first was the level set by the teachers, as listed in the KAS Book. The second was the frequency score (0 to 6) and the third the priority score (0 to 4), both determined by the study participants. The initial comparison was made between the opinion of the teachers (skill level) and the perception of the doctors (frequency and priority scores) using correlation coefficients (Table 2). Because all 797 respondents gave frequency and priority scores for all 114 skills, the sample sizes were sufficient to calculate correlation coefficients.

**Table 2:** Correlation among skill levels in the KAS book, frequency of use and priority

		Correlation coefficient between					
Kind of respondent	Number ofdoctors	Skill level in KAS book and frequency score by doctors		Skill level in KAS book and priority score rated by doctors		Frequency score and priority score rated by doctors	
		
		n	**r**	n	**r**	n	**r**
All Doctors	797	89622	**0.17**	89362	**0.15**	88978	**0.53**

General Medicine	274	30771	**0.16**	30727	**0.14**	30550	**0.47**
Internal Medicine	196	22061	**0.13**	21966	**0.14**	21892	**0.56**
Surgery	143	16141	**0.30**	16045	**0.23**	16011	**0.61**
Public Health	24	2268	**0.06**	2657	0.03*	2647	**0.52**
Others	160	17981	**0.11**	17967	**0.11**	17878	**0.52**

* p = 0.13. All other p < 0.001

Both the frequency and priority scores given by working doctors in each of the five groups were positively correlated with the skill levels set by the teachers. With the exception of the Public Health group, all correlation coefficients were statistically significant, though some correlations were weak. The correlations were strongest in the clinical groups, especially Surgery. The relationships between the frequency and priority scores were also strong, as might be expected if skills that are used more frequently would have higher priority. There were, however, still mismatches between skill levels and frequency and priority scores; these required more detailed examination.

### Frequency of using selected skills according to discipline

The frequency scores given by the 797 respondents are shown in Table 3. Doctors working in different specializations at different levels most frequently used skills belonging to the Basic and Infectious Diseases groups (means of scores were more than 3, used at least 1–2 times per month), while the skills in Parasitology, Traditional Medicine and Environmental Health were used less than 1–2 times per year (means of scores less than 1). The figures in the percentile distribution columns show how many of the respondents used different types of skills with different frequencies. For example, 50% of the respondents used skills in Basic and Infectious Diseases 1–3 times per week (P50 score 4), while for the last nine disciplines, only 5% used those skills with high frequency (P5 score 4). The frequency of using skills from Environmental Health was the lowest – the P50 was 0 and the highest score was still only 3.

**Table 3:** Frequency of use of selected skills

*Skills belonging to discipline*	No. of skills	Frequency score (*Mean *± SD)	*Percentile distribution*			
			
			*P50*	*P25*	*P10*	*P5*
1. Basic	11	3.7 ± 1.9	4	5	6	6
2. Infectious Diseases	2	3.2 ± 1.9	4	5	6	6
3. Internal Medicine	4	2.6 ± 1.8	3	4	5	6
4. Nutrition and Food Safety	2	2.3 ± 1.6	3	4	5	6
5. Paediatrics	8	2.3 ± 1.8	2	4	5	6
6. ENT	8	2.2 ± 1.7	2	4	5	5
7. Surgery	12	2.0 ± 1.7	2	3	4	5
8. Obstetrics & Gynaecology	15	1.7 ± 1.9	1	3	5	6
9. Tuberculosis	3	1.6 ± 1.5	1	3	4	5
10. Odontostomatology	4	1.5 ± 1.5	1	3	4	4
11. Psychiatry	5	1.4 ± 1.4	1	2	4	4
12. Health Management	9	1.3 ± 1.5	1	2	3	4
13. Dermatology	2	1.2 ± 1.3	1	2	3	4
14. Ophthalmology	8	1.2 ± 1.5	1	2	3	4
15. Epidemiology	8	1.1 ± 1.3	1	2	3	4
16. Health Education	3	1.1 ± 1.3	1	2	3	4
17. Parasitology	2	0.8 ± 1.4	0	1	3	4
18. Traditional Medicine	2	0.8 ± 1.3	0	1	3	4
19. Environmental Health	6	0.7 ± 1.2	0	1	3	3

Total	114					

**Figure 3:**
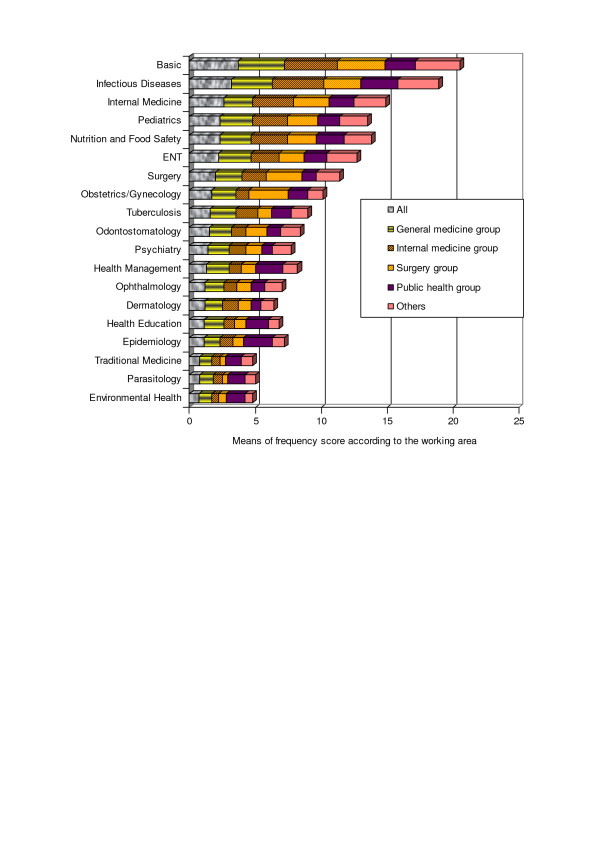
Frequency of skill use according to working areas.

When the working areas of the doctors were taken into account, we can see from the data in Figure 3 that even when working in different areas, doctors used the basic skills and those belonging to infectious diseases very often, even more frequently than skills in their own disciplines (see Surgery and Public Health groups). A number of skills (notably in dermatology, ophthalmology, parasitology and traditional medicine) were reported as used very infrequently by doctors working in all areas.

### Appropriateness of skill levels set by teachers compared to frequency of use by practicing doctors

In most cases, the practicing doctors' reporting on the frequency of using each skill was closely related to the level set by the teachers in the KAS book. In several cases, however, there were discrepancies (Table 4). For some skills, the teachers had set them at level 1 (can only do under supervision) but they scored high on frequency of use (mean score ≥ 2). In other cases, the teachers had set a high level (can do without supervision) for skills that the practicing doctors reported they seldom or never used (mean score < 1, P50 = 0).

**Table 4:** Discrepancies between set skill levels and frequency of use

Skills belonging to discipline	Name of skill	Skill level in KAS book	Frequency score (*Mean ± SD*)	Percentile distribution			
				
				P50	P25	P10	P5
Pediatrics	Giving medical instructions to monitor and take care of pediatric patients	1	3.0 ± 2.2	3	5	6	6
ENT	Diagnosis of acute otitis media	1	2.4 ± 1.7	2	4	5	5
Odonto-stomatology	Classifying soft tissue injury, identifying broken bones and combined injuries	1	2.1 ± 1.8	2	4	5	5
Tuberculosis	Diagnosing common types of tuberculosis	1	2.0 ± 1.5	2	3	4	5
Internal Medicine	Inserting stomach probe and doing gastric lavage	1	2.0 ± 1.8	2	3	4	5

Epidemiology	Calculating indicators related to community health, identifying health problems, variables	2	0.6 ± 1.0	0	1	2	3
Epidemiology	Selecting appropriate tools for collecting data	2	0.8 ± 1.2	0	1	2	3
Psychiatry	Conducting health education campaign for mental health	2	0.7 ± 1.2	0	1	3	3
Health Education	Making plan for health education campaign on a concrete health issue in community	2	0.8 ± 1.2	0	1	3	3
Environmental Health	Identifying clean water sources in a community.	2	0.9 ± 1.2	0	1	3	3
Traditional Medicine	Identifying acupuncture point formulas for treating eight common diseases and symptoms	2	0.8 ± 1.3	0	1	3	4
Traditional Medicine	Giving traditional medicine prescriptions for treating eight common diseases and symptoms	2	0.8 ± 1.4	0	1	3	4
Environmental Health	How to prevent and manage occupational fatigue.	2	0.9 ± 1.4	0	1	3	4
Parasitology	How to make blood slides to identify malaria parasites.	2	0.8 ± 1.4	0	1	3	4
Health Management	Analyzing information and indicators for health programs.	2	1.0 ± 1.3	0	2	3	3
Environmental Health	Guiding kindergarten teachers to use growth charts and monitor physical development of children.	2	0.9 ± 1.3	0	2	3	3
Ob-Gyn	Removing intrauterine devices	3	0.9 ± 1.5	0	1	4	4
Ob-Gyn	Insert speculum for genital exam.	3	1.5 ± 2.1	0	3	5	6

### Priority of the selected skills as perceived by practising doctors

The respondents were also asked to rate the priority of the 114 skills for their work, from 0 (no priority) to 4 (high priority). Basic skills and those skills belonging to internal medicine, paediatrics and infectious diseases were regarded as having moderate to high priority (mean of priority score ≥ 3 and four percentiles in the column 3 or 4). Skills in traditional medicine, the public health disciplines and several clinical disciplines were given at low priority (means of scores around 2) (Table 5). The distribution of these trends is similar to the distribution of the frequency scores shown in Table 3.

**Table 5:** Distribution of scores on priority of skills

*Skills belonging to discipline*	No. of skills	Priority score (*Mean *± SD)	*Percentile distribution*			
			
			*P50*	*P25*	*P10*	*P5*
1. Basic	11	3.2 ± 0.9	4	4	4	4
2. Internal Medicine	4	3.2 ± 1.1	4	4	4	4
3. Paediatrics	8	3.0 ± 1.1	3	4	4	4
4. Infectious Diseases	2	3.0 ± 1.0	3	4	4	4
5. Surgery	12	2.8 ± 1.1	3	4	4	4
6. Obstetrics & Gynaecology	15	2.6 ± 1.2	3	4	4	4
7. Nutrition and Food Safety	2	2.6 ± 1.0	3	3	4	4
8. Tuberculosis	3	2.6 ± 1.1	3	3	4	4
9. ENT	8	2.5 ± 1.1	3	4	4	4
10. Odontostomatology	4	2.5 ± 1.1	2	3	4	4
11. Ophthalmology	8	2.3 ± 1.1	2	3	4	4
12. Dermatology	2	2.2 ± 1.1	3	3	4	4
13. Psychiatry	5	2.2 ± 1.1	2	3	4	4
14. Epidemiology	8	2.2 ± 1.0	2	3	4	4
15. Health Management	9	2.1 ± 1.1	2	3	4	4
16. Health Education	3	2.1 ± 1.0	2	3	3	4
17. Parasitology	2	2.1 ± 1.2	2	3	3	4
18. Environmental Health	6	2.0 ± 1.1	2	3	3	4
19. Traditional Medicine	2	1.6 ± 1.1	2	2	3	4

Total	114					

### Discrepancies between skill levels set by teachers and priority rating by practicing doctors

In general, skills that teachers set at a high level of proficiency were also rated as having high priority by the practicing doctors, but there were discrepancies, as shown in Table 6.

**Table 6:** Discrepancies between set skill levels and priority ranking

Skills belonging to discipline	Name of skill	Skill level in KAS book	Frequency score *(Mean ± SD)*	Percentile distribution			
				
				P50	P25	P10	P5
Pediatrics	Giving medical instructions to monitor and take care of pediatric patients	1	3.2 ± 1.1	4	4	4	4
Internal Medicine	Inserting stomach probe and doing gastric lavage	1	3.0 ± 1.1	3	4	4	4
Odonto- stomatology	Classifying soft tissue injury, identifying broken bones and combined injuries	1	2.8 ± 1.1	3	4	4	4
Tuberculosis	Diagnosing common types of tuberculosis	1	2.8 ± 1.0	3	4	4	4
ENT	Diagnosis of acute otitis media	1	2.7 ± 1.0	3	4	4	4
Epidemiology	Identifying tests to diagnose Cholera, typhoid and dysentery	1	2.7 ± 1.1	3	4	4	4
Health Management	Organizing and implementing measures for infection prevention in hospitals	1	2.6 ± 1.2	3	3	4	4
ENT	Diagnosis injury in ear, nose, throat	1	2.5 ± 1.1	3	3	4	4

Traditional Medicine	Identifying acupuncture point formulas for treating eight common diseases and symptoms	2	1.6 ± 1.1	2	2	3	4
Traditional Medicine	Giving traditional medicine prescriptions for treating eight common diseases and symptoms	2	1.6 ± 1.1	2	2	3	4
Epidemiology	Calculating indicators related to community health, identifying health problems, variables	2	1.8 ± 1.0	2	2	3	4
Health Management	Analyzing information and indicators for health programs.	2	1.8 ± 1.1	2	2	3	4
Psychiatry	Conducting health education campaign for mental health	2	1.9 ± 1.0	2	3	3	4
Health Management	Producing report on health information and statistics of district and commune health center.	2	1.9 ± 1.2	2	3	4	4
ENT	Identifying normal and abnormal status of nose.	2	1.9 ± 1.1	2	3	3	4
Health Management	Applying concrete solutions to implement health policies	2	1.9 ± 1.1	2	3	3	4
Epidemiology	Selecting appropriate tools for collecting data	2	2.0 ± 1.0	2	3	3	4
Environmental Health	How to prevent and manage occupational fatigue.	2	2.0 ± 1.1	2	3	3	4
Ob-Gyn	Removing intrauterine devices	3	2.0 ± 1.2	2	3	4	4
Ob-Gyn	Insert speculum for genital exam.	3	2.3 ± 1.2	2	3	4	4
Ob-Gyn	Guiding how to use condoms	3	2.4 ± 1.1	2	3	4	4
Ob-Gyn	Counseling on oral contraceptives	3	2.4 ± 1.1	2	3	4	4

The list of discrepancies includes skills that the teachers set at level 1 (can do only under supervision) but were rated moderate priority (mean score around 3) by the practicing doctors, suggesting that they should be able to do them at a higher level. Other skills were set at the higher levels 2 and 3 by teachers (can do without supervision) but were considered to have low priority by the practicing doctors.

### Focus group discussions

During the focus group discussions (FGD), the clinical doctors working mainly in hospitals emphasised that they had not been well trained in skills such as combining the information from all sources to produce a differential diagnosis, or good decision-making skills, particularly in emergency situations. Communication skills, including taking patient history and communicating effectively with colleagues, were also mentioned as being inadequately developed during their training. The doctors also thought their knowledge and skills in basic subjects especially related to laboratory tests were weak and could be improved by better and more integrated teaching of clinical and basic sciences.

"We still lack skills on how to deal with emergency cases especially for surgery, and communication skills including the communication and working in a team with other colleagues in both higher level, or in other sectors." And, "We lack confidence in front of the patients – we found that some cases when we refer to higher level is not because of the medical need but the family demands transfer and we are not confident enough to insist that we can provide the needed care ourselves, so we follow the patients' and families' requests." *(both from FGD with doctors in curative sector)*

The doctors in preventive medicine reported that they had not been trained well enough in skills of planning, health management information systems, data collection, analysis and reporting. They had failed to acquire communication skills appropriate for public health, such as the communication with the community and especially with other sectors to improve coordination for health interventions. Doctors working at district but not provincial level needed more of the basic technical skills such as collecting samples for water and food safety tests. These doctors also thought they lacked skills necessary to identify and report outbreaks of diseases.

"We lack skills in supervision and in collecting and analyzing data and presenting it in reports; we also lack communication skills, and don't know how to conduct heath promotion campaigns. Because we do not have enough skills in planning and in collecting and using information, we have to accept the plan from the higher level. "*(FGD with doctors in preventive sector)*

Doctors in both clinical and preventive groups noted that they had not received sufficient training in the many laws that regulate not only medical practice but also labour and civil law applying also to administration and management. They also needed more preparation for their roles in training and supervising colleagues at lower levels in the health delivery system.

From all of the discussions, the respondents proposed 45 skills that they thought were necessary, but were not mentioned in the list of 114 skills in the data collection form. However, only 21 of the 45 skills (Table 7) were new; the other 24 were included in the KAS book but had not been presented in the survey.

**Table 7:** Additional skills suggested by practising doctors during FGD

**A. Clinical (6 skills)**
1. How to hold consultations to get agreement on diagnosis and treatment patients
2. How to evaluate vital functions of a patient
3. How to follow up patient treatment at home
4. How to check quality of patient records
5. How to establish regulations for working and being on duty in hospital
6. How to guide people to use traditional medicine
**B. Preventive (8 skills)**
7. How to conduct an in-service training course
8. How to communicate effectively with poor and/or minority patients
9. How to check and monitor patient and health records
10. How to direct implementation by lower levels
11. How to make a good family visit
12. How to manage human resources, facilities, money and health information
13. How to manage projects
14. How to summarize the reports from lower levels to get good evidence for planning and management.

**C. Laboratory (1 skill)**
15. How to summarize, analyze and interpret results of laboratory tests

**D. Others (6 skills)**
16. How to assign tasks and responsibilities to staff in a team
17. How to apply results from research into practice
18. How to organize a scientific seminar or a symposium
19. How to find and read foreign literature
20. Self-learning skills
21. How to learn from older colleagues

## Discussion

Up to 1994, teaching in medical schools in Vietnam was hospital centred as well as teacher centred; the curriculum was reviewed and revised using only the experience of the leaders in the medical schools and ministries. During the past ten years, the emphasis has been shifting to train community-oriented doctors rather than hospital-oriented doctors, and to a revision process that includes collecting data from different points of view.

This study was based on the model of the Johari Window, which is often used to analyse the perceptions of individuals but can also be used to analyse organisations [7]. The window image has four panes, representing four levels of shared information. The first is the open or public area, containing information known by one's self and by others, the second a blind area, visible to others but not to one's self, the third is the hidden area, known to the individual but not to the outside, and the fourth holds the unknown, information that neither the self nor others perceive. An important application of this model is to illustrate how getting appropriate feedback from other stakeholders can reduce the blind area and enlarge the public area. In this study, feedback from recently graduated doctors working at different levels in different specializations about the learning objectives developed by the teachers made it possible to identify appropriate and realistic skills needed by doctors working in the community. This gave the teachers an expanded view of the real needs of their students, both confirming what they had assumed to be in the public window and increasing the size of the window that had been visible to others but not to them. The importance of obtaining information from a range of stakeholders to develop an appropriate medical curriculum was emphasized by Snell, Tallett, Hajat, Hays, Norcini, Prince, Rothman & Rowe [8]. Reviewing methods to evaluate a curriculum, they concluded that it was best to make a connection between teaching and learning and the expected function of a physician, to lead to the improvement of student learning and the achievement of better health care practice.

The study sample can be considered as representative for doctors working in the public sector around Vietnam, which is most of the practicing doctors. The schools selected the 16 provinces (out of 64) randomly, and in each province, most of the eligible subjects were recruited for the study. The distribution of areas of work among the respondents was similar in every province surveyed.

The main question concerned the appropriateness of skill types and levels set by the teachers in the book of learning objectives. The results show that in most cases, the practising doctors agreed with what the teachers had proposed as learning objectives for their graduates. The criteria applied for the relevance of the skills and levels were the frequency with which the doctors used the skills and the priority they assigned to each skill, assuming that high priority, frequently used skills should be taught to a higher skill level. In general, the correlations between the skill levels set by the teachers and the rating by practising doctors both for frequency for were highly significant, except for doctors working in public health. This difference can be explained by the low number of respondents working in this field (24), while most of skills listed in the data collection form were more appropriate for clinicians.

There was great variation in the frequency of use of skills belonging to the different disciplines. For example, skills in infectious diseases and in nutrition and food safety were used frequently by many doctors, showing that these are still common health issues at every level in Vietnam. Concerning skills of clinical disciplines that were infrequently used, for example in dermatology, it may be that few respondents were working in areas where those skills are most often needed; those disciplines are also allocated less time in curriculum. The low frequency of using the skills of public health disciplines (health management, health education, epidemiology, environmental health) is probably due to the bias in the study population towards clinical doctors. Although doctors more often used the skills belonging to their own group, the skills classed as general and those from infectious diseases and internal medicine were used frequently by all groups. This information needs to be considered when revising the KAS Book developed by the teachers.

Although the selection of the skills for the survey was based on the teachers' assessments of frequency of use and priority for learning, there were some notable differences of opinion and/or experience. Some skills were set at a low level by the teachers but given a high score by the young doctors and vice versa. Since we did not ask about all of the level 2 and level 1 skills, there may be more of these mismatches between teaching and practice among the skills not included in the survey. In preparation of the next revision of the book, additional surveys among practicing doctors will be needed to cover all skills so that the required levels can be set according to real needs in practice.

Other skills, which were listed by teachers as being needed at a lower level, were reported as being used often by the practising young doctors. Perhaps these skills should be set at level 3 in the book of learning objectives and should receive sufficient attention in the curriculum to ensure that students learn them thoroughly before graduating and going into practice.

During group discussions, the doctors also proposed that several skills that were not taught in medical schools and not listed in the KAS Book were necessary for newly graduated medical doctors. These suggestions were important for the revision of the book.

It is important to note that most doctors in Vietnam will not work alone after graduation, but will work in a team with experienced health staff, even at grassroots levels and will be absorbed into the national health system where they will usually work with senior and more experienced colleagues from whom they will also learn to improve their skills. Therefore, one issue that should be taken into consideration is whether teachers should set high level for skills that young doctors use very often after graduating or just teach them with a basic level and let them be improved by practicing when the young doctors work at the field. That would require a system to monitor and evaluate the skills development in the years after graduation and perhaps a more structured preparation of the supervising doctors in their task of coaching the new graduates – something like residency systems used in other countries.

The focus group discussions provided additional suggestions about skills that were not included in the survey but were considered to have moderate priority by the clinical and public health doctors or both. These were mainly not technical skills belonging to a particular department but the cross-cutting skills such as decision-making and communication with colleagues that may have been ignored because in a discipline-based curriculum, no department was responsible for them. The need for skills in combining information to solve problems and make decisions and the lack of preparation for that was noted by the clinical doctors. This may reflect the curriculum structure in Vietnamese medical schools up to now which is discipline based and not problem based. The new learning objectives were, however, identified on the basis of problems and a start has been made on introducing problem based learning during the last few years.

The doctors working in preventive medicine noted many skills that they felt did not get enough attention during their medical training. That is one reason that the MoH in Vietnam is now planning to introduce a new training track in the medical schools that will focus more on the skills for preventive medicine, with graduates who are doctors of preventive medicine.

The findings our study are comparable in overall results to those from other surveys carried out as part of evaluation of an existing, changed or proposed curricula. For example, Blumenthal, Gohkale, Campbell and Weissman [3] reported that a national survey of residents of all specialties in the United States showed that they rated themselves as well prepared by their training, but more than 10% also reported that there were tasks they should do but could not do, indicating opportunities to improve their training. When graduates of a Canadian general internal medicine training program were asked whether their training fit their needs once they were working, areas were identified that needed more attention in the training [9]. Evaluation of a curriculum may be carried out through questionnaires designed against a set of nationally agreed standards. Using this approach, Jones, McArdle and O'Neill [10] surveyed the perceptions of graduates on new and old courses at Manchester University, UK, and found that across a range of broad competencies and specific skills, graduates of the new course considered that they were better prepared for their professional practice.

## Conclusion

The results presented in this paper suggest that the participatory approach used to develop the revised curriculum for Vietnamese medical schools produced priority areas that seem to concur with relevance to the daily practice of the graduates, although a number of important corrections were also identified that should be brought into the next version of the learning objectives. The usefulness of the contributions from practicing doctors to development of appropriate learning objectives was confirmed.

## Competing interests

The author(s) declare that they have no competing interests.

## Authors' contributions

HNL was the project coordinator; he guided the design, implementation and analysis of the survey, carried out statistical tests and drafted the manuscript. DVD was the team leader for the project in his faculty and led the design and data analysis for the survey, contributed to the statistical analysis. EPW was technical adviser to the project, and contributed to the design and planning of the data collection, and to data analysis and drafting the manuscript. All authors read and approved the final manuscript.

## Pre-publication history

The pre-publication history for this paper can be accessed here:


